# On-the-Fly Formation of Polymer Film at Water Surface

**DOI:** 10.3390/polym14153228

**Published:** 2022-08-08

**Authors:** Veronica Vespini, Sara Coppola, Pietro Ferraro

**Affiliations:** Institute of Applied Sciences and Intelligent Systems “E. Caianiello”, National Research Council (CNR-ISASI), Via Campi Flegrei 34, 80078 Pozzuoli, NA, Italy

**Keywords:** film, interface, liquid packaging, Marangoni propulsion, polymer, spreading, surface tension

## Abstract

The self-propulsion of bodies floating in water is of great interest for developing new robotic and intelligent systems at different scales, and whenever possible, Marangoni propulsion is an attractive candidate for the locomotion of untethered micro-robots. Significant cases have been shown using liquid and solid surfactants that allow an effective propulsion for bodies floating on water to be achieved. Here, we show for the first time a strategy for activating a twofold functionality where the self-propulsion of a floating body is combined with the formation of a polymer thin film at the water surface. In fact, we demonstrate that by using polymer droplets with an appropriate concentration of solvent and delivering such drops at specific locations onto freely floating objects, it is possible to form “on-the-fly” thin polymer films at the free water surface. By exploiting self-propulsion, a polymer thin film can be formed that could cover quite extensive areas with different shapes depending on the motion of the floating object. This intriguing twice-functionality activated though a single phenomenon, i.e., film formation and related locomotion, could be used in perspective to perform complex operations at water surfaces, such as dynamic liquid packaging, cleaning, and moving away floating particles, monolayer films, or macro-sized objects, as discussed in the text.

## 1. Introduction

Sensing, imaging, microfluidics, cargo manipulation, high-precision delivery, biomedical prosthesis, and environmental remediation are just a few sectors that show a strong interest in autonomous, self-propelled, and self-powered nano-, milli-, and micromachines [1,2,3,4]. Thus far, numerous approaches for self-propelled micromotors have been developed but are often based on sophisticated designs [5,6,7] and require complex numerical simulation models for the analysis of motion [8,9]. The interest in controlling locomotion is also related to the powerful potential application in biomedicine, including active drug delivery, precise cell manipulation, noninvasive surgery, biosensors, and imaging in vivo, while induced motility is also a key motivation in the domain of artificial cell design [10]. It has recently been demonstrated that self-propulsion considerably enhances the assembly of polymers: self-propelled molecules are found to assemble faster into polymer-like structures than non-self-propelled ones [11]. Even if it is desirable to develop autonomous systems, able to adjust their own trajectory based on the collection of information from the surrounding medium, a remarkable goal would be the simplification of the self-propulsion mechanism. Referring to the biological world, the mechanism used by the water strider Microvelia and by some terrestrial insects that accidentally fall into water could be very interesting and inspiring to duplicate [12]. Water walking is, in fact, a spectacular means of locomotion that nature has improved with some aquatic insects (i.e., Stenus and Velia) that can run away from the water surface by easily secreting chemical material, which temporarily lowers the surface tension behind them. These insects practically induce locomotion by surface tension gradients by excreting “on the-fly” a surfactant that allows them to accelerate the motion on the water surface. Such a surface tension gradient can be generated by modification of the surface the object moves on, and such locomotion is commonly known as Marangoni propulsion [13,14]. The Marangoni effect is a mass transfer phenomenon between two fluids with different surface tensions, such as a solvent in water. More generally, the modification of liquid surroundings by an object deposited on them can occur not only by a chemical reaction but also by diffusion, spreading, and solute mass transfer. The main advantage of the liquid substrate sustaining motion is the enhanced possibility of fast regeneration of the surface after the object has passed at the time scale of the movement, allowing motion on a circular trail or periodical deformation. The first experimental investigation of the Marangoni effect starts with the self-propulsion of a micro-sized object, such as a camphor boat [15,16], and a new class of polymer gel that undergoes translational and rotational motion in the water [17]. Starting from this result, it has been demonstrated that a floating object (typically in the shape of a boat in polystyrene foam) can be propelled along the water surface, and the direction of the motion can be influenced by the boat design. Different studies have focused on the kinds of “fuels” that could be employed regarding safety, effectiveness, duration, case of use, and availability [18]. The possibility to use a camphor disk as a motor to propel a small boat made of a polyester sheet has also been addressed. When such a disk is stuck at the stern of the boat, directional motion is observed for a few tens of minutes in a circular canal [19], while intermittent motion is obtained by simply changing the position of the camphor motor [20]. Very recent studies showed that it is possible also to control the self-propelled motion of a camphor disk by manipulating contributing factors at the molecular level [21]. More complex experiments have been explored by combining the motion properties with a case of use. In fact, macro-sized motors propelled by the Marangoni effect have exhibited fast and long-range motion, thus representing good candidates to execute the tasks of environmental pollution detection and remediation in real-world situations (i.e., river or lakes) [22]. The duration of self-propulsion is manly related to the amount of solvent in the tank. Alternative approaches involve nanocellulose aerogel membranes that are permeable to gases but impermeable to liquids (water and oil), thus allowing locomotion and flotation on the surface [12]. Alternative strategies demonstrate passive directional motion without an active controller obtaining thrust by periodically dripping alcohol behind to utilize the Marangoni effect, but more complex components and design are needed [23]. Recently, self-induced motion has been widely studied for two main cases. The first is a system for which the propellant container is the propelled object itself, or a system where the propellant reservoir is embarked on manufactured floats from which the directional release of the spreading liquid is the motion-determining parameter [24]. Second, a robot travelling on water based on a Marangoni propulsion system has been demonstrated using a flow-imbibition-powered microfluidic pump with a magnetic clamping to trigger the formation of alcohol droplets at required times [25]. The results obtained are very impressive but still require advanced technological expertise. A different and more simple approach, proposed by Zhao et al. [26], consists of solid polymer capsules on water, where the Marangoni effect has been employed for the autonomous motion of a millimeter-sized capsule motor at a liquid/liquid interface, while the movement control is operated through an external magnetic field on different media [27]. The same group demonstrated a self-driven millimeter-sized polymer capsule that could move at a very high velocity on a wide variety of liquid/air interfaces, where the mechanism of their motion was mainly based on the Marangoni effect, whereby objects move from a place of lower surface tension to a place of higher surface tension [26]. Alternative applications of the Marangoni effect are still being tested, including the results of Marangoni propulsion micro-robots integrated with a photonic gel sensor for exploring aquatic environments; these results open a new route for green chemistry [28]. Marangoni propulsion micro-robots integrated with a wireless temperature sensor have very recently been used for sensing external temperature, while the micro-robots propel at the water–air interface [28]. Very recently we demonstrated a new working principle for liquid locomotion and solid manipulation by using a multipurpose platform guided by a pyroelectric platform [29,30,31,32]. Malkin and coworkers focused on the spreading of a polymer solution at the interface, leading to a transition from a three-dimensional structure to a two-dimensional film [33]; they investigated the spreading kinetics of multicomponent solutions with different concentrations over a water surface, measuring the two-dimensional rheological properties of the formed film obtained by phase separation [34,35]. The film formation on liquid induced by phase inversion was mainly employed in the quick liquid packaging process we proposed for the easy fabrication of two- and three-dimensional polymeric film on the water surface [36]. The thin films were formed in a static configuration: a polymeric solution was delivered in a single shot on liquid, thus enveloping all the volume and perfectly accommodating its 3D contour. The idea of combining the liquid packaging approach with the Marangoni-propelled micro-robots guided us to the development of the innovative and intriguing functionalities revealed and discussed in this work. In fact, here, we show a new approach where a single polymer drop could induce the Marangoni-driven self-propulsion of common macro-objects floating on water and, at the same time, could drive formation “on the fly” of a thin polymer film at the water surface.

The innovation consists of adopting a process where the same solvent used for the preparation of the polymeric solution and the film formation is employed at the same time for the creation of a surface gradient able to induce the self-propelling of the object. Controlling the position where the droplet is delivered and the total amount of the dispensed volume, it is possible to tune the size, shape, and extension of the formed film. It is important to underline that the film thus formed floats on the surface of the water and can extend tens of centimeters. One great advantage of the new method proposed is that it would be possible to dynamically form the thin films through a very simple modality, i.e., without spending time and resources to design micro-objects, but rather controlling their locomotion through Marangoni propulsion. In the following, a detailed description of the film formation is illustrated in combination with the object propelling and the description of some intriguing functionalities. The reported results can allow one to foresee that such a polymer thin film could be used for multiple purposes, such as, for example, cleaning a specific site by moving away a monolayer film and floating particles or a macro-sized object. The method is electric-free, instantaneous, independent of water surface condition, and noise-free and no unnecessary exhaust products, such as combustion or other chemical reactions, are released.

## 2. Materials and Methods

### 2.1. Materials

Poly (lactic-co-glycolic acid) PLGA (38,000 to 54,000 Da; PLGA 504 H, Boeringer Ingelheim, Ingelheim am Rhein, Germany) was dissolved in dimethyl carbonate (DMC 20–30 *w*/*v*, DMC 99%, Sigma-Aldrich, St. Louis, MO, USA). In all the experiments described in the following, the concentration of the PLGA/DMC solution was set at 25% *w*/*w*, and Nile Red was added to make it easier to work with the solution, which otherwise appears transparent. The solvent selected and the concentration of the polymeric solution were fixed; in fact, we tested the property of the as-formed thin films in terms of mechanical, permeability, and gas barrier properties as well as optical transmittance [36]. The experiments were carried out at room temperature, and no chemicals were added to the water to tune the surface tension. Pyrex glass beaker (size 500 mL) and plastic container (size 40 × 60 cm) were filled with tap water and used for propulsion experiments.

We used Tracker.jar for velocity and trajectory analysis in the Open-Source Physics (OSP) Java framework. This allowed us to import the video of propelled objects and to track the motion of more than one point on the object of interest frame by frame. The data acquired were elaborated to track displacement, velocity, and acceleration.

### 2.2. Principles of Propulsion and Film Formation

A polymeric solution drop of PLGA/DMC when delivered on the free water surface can instantaneously form a continuous and thin polymeric film [36]. Alternatively, when the PLGA/DMC drop is delivered in a very small, closed compartment, the film cannot form due to the absence of the available water surface for spreading all over. However, it is well known that when a solvent droplet is facing the water, the self-propulsion mechanism can start, as the solvent is diffused in the water [12]. Thus, in our case, the release of solvent in the water drove the propulsion as usual, but the phenomenon was coupled with concurrent polymer thin film formation at the interface. The combination of these two effects, consisting of delivering a solution drop, for example, at the rear of a floating object (i.e., mini-boat) and inducing the film formation “on the fly” guided by the self-propelled floating object, is described here for the first time.

**Figure 1:**
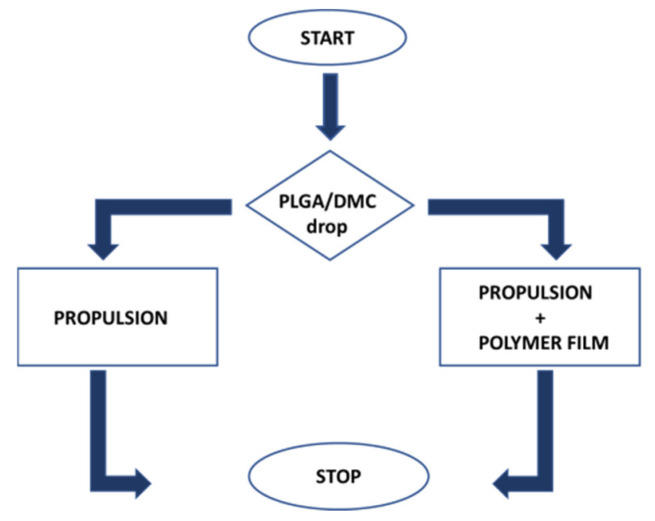
Flow chart of the process related to on-the-fly formation of polymeric thin films at water surface.

**Figure 2:**
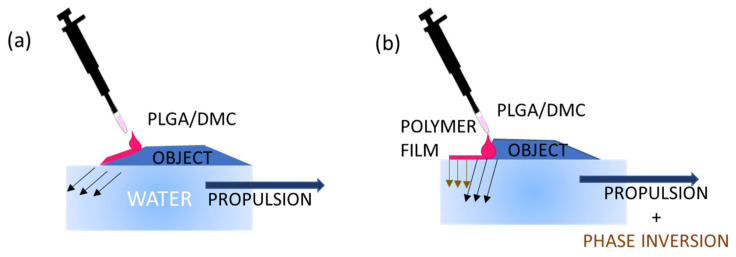
Scheme illustrating the mechanism of release of polymeric solution drops on two different side geometries. (**a**) Sloped side for slow release: the droplet is released along the oblique side wall so that only a small volume will be in contact with water, missing the free air–water surface needed for spreading, and as a consequence, only the propulsion mechanism is activated. (**b**) Almost perpendicular wall for fast release: the droplet is released just on top of the side and, once dispensed, will immediately and entirely face the water surface, activating film formation and Marangoni propulsion at the same time.

The mechanism of propulsion and film formation can be described in successive steps, and the corresponding flow-chart is reported in Figure 1. The first one is the creation of surface tension gradients by the leakage of the solvent in the water. However, if the droplet is positioned in a location where it can face a limited area of free air–water surface, as depicted in Figure 2a, only the propulsion is activated without film formation. This can also occur in a situation in which the droplet is dispensed in the proximity of the edge where just a small surface of the polymer drop is facing the water. Once the dispensed drop comes into contact with the water, the DMC is released from it and rapidly spreads by diffusion. The diffusion of the organic solvent on the aqueous surface imparts the reaction force to the objects that thus drives the one-directional propulsion of the floating object. Essentially, the release of the solvent compound induces a surface tension imbalance, and then, due to anisotropy of the object itself, it is possible to observe the start of a motion, gently pushing the object in clear direction, as in the case of the camphor boat [19,20]. When a large portion of the droplet surface comes in contact with the water surface (for example, by a drop slipping toward the free-water surface), the polymer is forcefully and widely expanded by the violent solvent extraction (see Figure 2b). Thus, the thin polymer film takes place at water surface.

The formation process of the thin film happens in a fraction of second, and it is induced by phase inversion under solvent diffusion. At the same time, upon interaction with water, in the last step, the PLGA molecules solidify, creating a continuous polymeric film that can reach several centimeters in length. The explosive spreading and the kinetics curves for different polymer concentrations have been extensively studied in the case of a multicomponent drop delivered on water surface [35]. The main difference with the experiments proposed in the following consists of the radial shape of the film produced in the static condition, i.e., when the drop just falls into the water. In our case, the polymeric film could assume a different shape depending on the use of Marangoni-propelled micro-objects.

As a result of the above steps, the floating object is accelerated and propelled. The mode of motion is determined by whether or not the net reaction force passes through the mass center of the object. Once the motion is initiated, the film is formed “on the fly” over the water surface until the object is stopped. The motion is sustained only if there is a physico-chemical process restoring the free surface after the passage of the object. The extended investigations performed by Nakata’s group have established the dependence of motion on several parameters, such as chemical structure of the material, surface tension of the supporting liquid, friction originating from the aqueous phase, shape of the cell, and the shape of the scraping [36].

**Figure 3:**
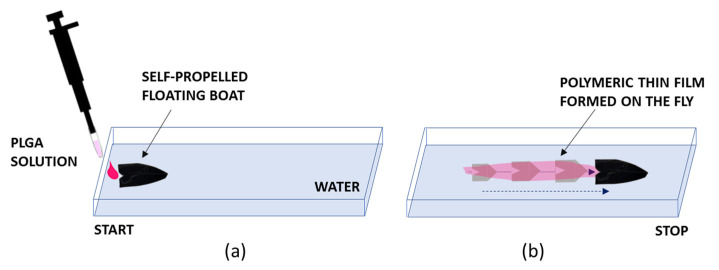
Scheme illustrating the mechanism of self-propulsion by the solute capillary Marangoni flow. (**a**) Dispensing of the polymeric solution for propelling the object (a black boat). (**b**) Propulsion of the object and simultaneous formation of a polymeric film along the direction of the propulsion. The object while moving over the water surface activates the formation of a thin film by polymer spreading on the water surface.

The movement of the object was driven by the asymmetric release of DMC from the drop via an interlayer Marangoni effect. The volatility of the gaseous Marangoni fuel would enable the restoration of the original surface tension of the carrying liquid once the object passed this location. In fact, the dispensed drop released DMC, which is miscible with water. Since DMC exhibits a much lower surface tension (γ = 28.55 mN m^−1^) than water (γ = 72.0 mN m^−1^), the object could be pulled toward the direction of the solution with a higher surface tension [20]. As the solvent passed asymmetrically in the aqueous phase, the difference in surface tension at the opposite side of the object resulted in the motion of the object. Alcohol evaporated and, due to the shape of the boat, created the area of lower surface tension at the rear side of the boat, pushing it forward. The DMC solvent was selected here, focusing on the future prospective of biomedical applications of the as-formed films. The formation of a thin polymeric film on the fly was induced by the quick polymer spreading process (Figure 3). In fact, due to the solvent extraction from the solution droplet by the water and the spreading forces due to interfacial Marangoni tension, a transparent and homogeneous thin film readily formed over the free water surface in the same direction of the propelled path. Of course, the shape of the object also has a role in the locomotion, but we show in the following that not only boats but essentially any object can be propelled quite easily using the method proposed. In fact, in the following section, we will describe the activation of self-propulsion and thin film formation on the fly for different objects, going through their implementation in detail.

## 3. Results

### 3.1. Polymeric Thin Film Formed on Water

**Figure 4:**
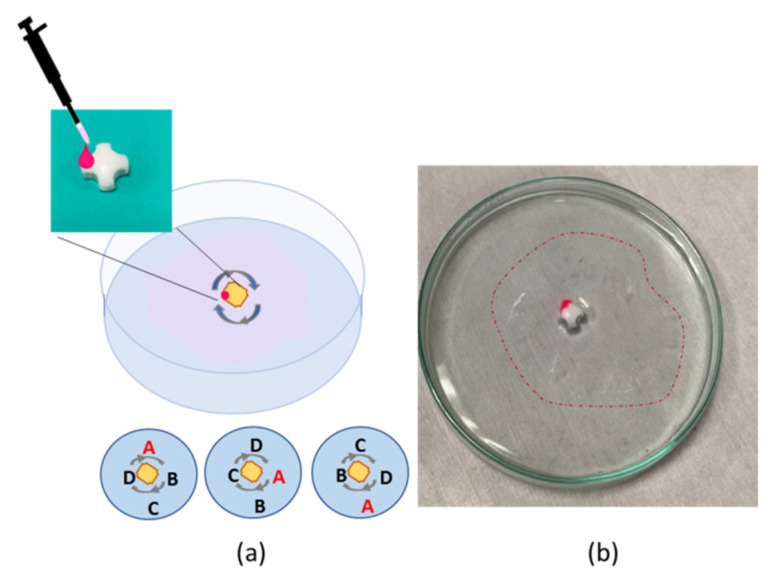
(**a**) Outline of the experiment: the white magnet commonly used for stirring is activated by the dispensing of polymer droplet, and the rotation of the magnet induces the film formation. (**b**) The polymeric film formed on the fly is indicated by the dotted line.

The self-propulsion by Marangoni flow is here adopted for the no-contact movement of common objects that, during their propulsion, can form a thin polymeric film on the fly. We tested polymeric lightweight as well as metallic objects, free to move or anchored in space. We pointed out that using the method proposed, there is no special constraint on the selection of the material or regarding the geometry of the object used, and no effort in designing and functionalizing it. The polymeric solution was delivered on the object of interest by simply pipetting polymer droplets of about 8 μL each. We started the experimental characterization using a heavy object, a commercial magnet usually employed for stirring. The white magnet was placed at the bottom of the Petri, but it was not totally covered by water, and the polymeric drop was delivered on the upper left side. Once the drop came into contact with the water, the object started to self-rotate, and a very thin and transparent film was formed until the rotation stopped. The film, in this case, assumed a spiral shape because of the rotational motion activated, keeping the magnet fixed in space but free to rotate. Even if the polymeric solution was colored by adding a fluorochrome—in fact, the solution appeared pink at the tip of the pipette—the small amount used for the activation of the self-rotation and film formation brought to the formation a transparent thin layer. The film’s formation process on the fly is clearer in Supplementary Video S1; in fact, the dynamic evolution can help the reader to detect and observe the floating film while forming. In Figure 4, a red dotted line is used to point out the area where the as-formed thin layer was lying.

**Figure 5:**
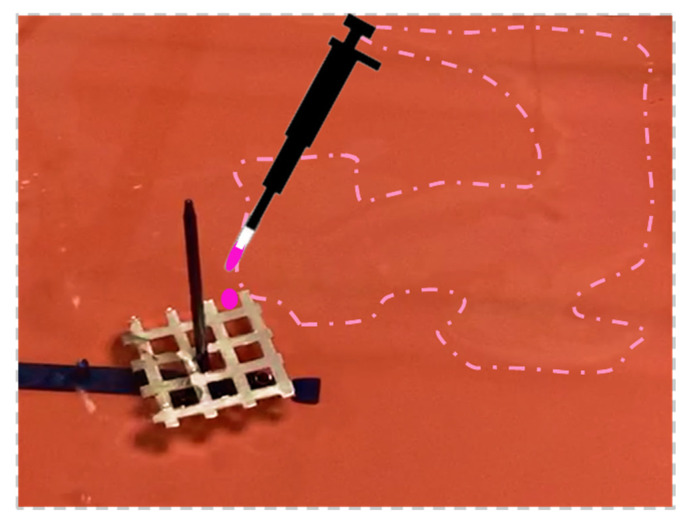
Al square grid forms an extended polymeric layer; the expanded film stops the grid’s rotation. On-the-fly formed polymeric film is indicated by the dotted line.

If the object floats on water and it is constrained with a fixed axis, the self-propelled Marangoni rotation can start using as a rotational axis the fixed one, and a polymeric film can form on the fly (Supplementary Video S2). The floating object selected in this case was an aluminum (Al) grid. A square Al grid was introduced in a rectangular plastic container filled with tap water and fixed with a pivot passing through its central spot, equidistant to the vertexes. The polymeric fuel droplet (8 μL) was delivered to the external spot (top right), activating the Marangoni flow self-rotation along the predefined central axes. While the self-propulsion was taking place around the pivot, it was possible to observe the thin film forming on the fly following the locomotion path of the square grid, as shown in Figure 5; once the film was formed, the movement ended. The direction of rotation could be tuned by dispensing the polymeric droplets in different locations.

**Figure 6:**
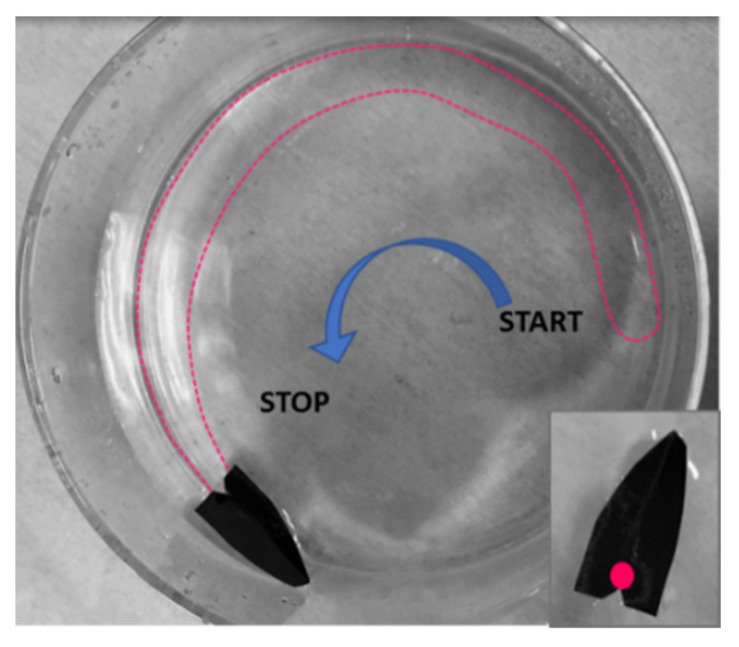
The dispensing of the polymeric droplet over the black boat floating on water induces the propulsion along the wall of the Becker and the simultaneous formation of a very thin semi-circular polymeric layer. On-the-fly formed polymer film is indicated by the dotted line.

Additional experiments were conducted using lightweight floating objects free to move over the water surface, such as in the case of a commercial plastic tube (length: 1 cm; diameter: about 1 mm). The tube was floating over the water surface in a Petri dish, while the polymeric drop was dispensed on its external longitudinal slide. As visible in Supplementary Video S3, the tube started to move towards the glass wall while the polymeric film was formed on the fly at the back. Even if the tube was stopped by the wall, the propulsion continued until the solvent was completely released in water, followed by the film formation on the fly, as visible by the wrinkling arising along the borders. In the former experiment, the process was stopped because of the dimension of the container selected, but using a container as large as 40 cm in length, the entire process could also involve linear locomotion. In order to study the effect of the container on the object’s propulsion, we adopted a very simple boat made of a metallic folded substrate totally floating on water as the model object and compared the results of its propulsion in a circular Becker and in a plastic rectangular bowl. In both the experiments, the polymer droplets were deposited in the rear back of the boat. In the case of propulsion in the Becker, the boat started to move along the glass slide, as visible in Figure 6 and Supplementary Video S4.

**Figure 7:**
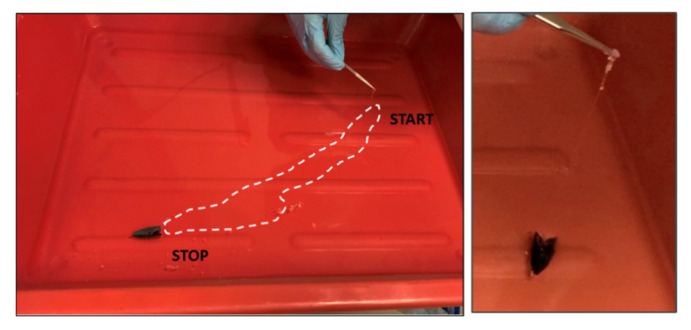
When the boat is free to move over a bigger surface, we observed the propulsion from one side to the opposite one. On-the-fly formed polymer film is indicated by the dotted line. The recovered polymeric film attached to the back of the boat is visible.

While the boat was moving along the side, a polymeric film was formed following the boat direction until the solution ran out. The process of propulsion and polymer spreading with film formation was also tested in the case of the bigger bowl, where the boat could propel linearly from side to side just by dispensing one polymer drop. During the movement, it was possible to observe the formation of the polymeric layer that, as in the previous case, remained anchored to the rear part of the boat. The polymeric film acted as a sort of tether, as visible in Figure 7, while in Supplementary Video S5, it is clearly visible how it is possible to move the boat for recovering the formed film.

From the experimental results, we can assume that the polymeric drop acted as the fuel of Marangoni locomotion. In synthesis, when the polymeric solution droplet was dispensed on the rear back of the object and not constrained in a delimitated area, the locomotion process could start. The spreading of the polymeric solution on the free water surface formed a thin and transparent layer. Once the film was formed, the object remained blocked within it, and the propulsion stopped, as clearly visible in the Supplementary Videos. If the transparent polymeric layer is recovered, the propelled object can be transported within. The velocity and the time of the locomotion are a function of the total amount of solution delivered and also depend on the concentration selected for the solution. By pipetting additional drops, it would be possible to restart the combined process.

### 3.2. Velocity and Trajectory Analysis

**Figure 8:**
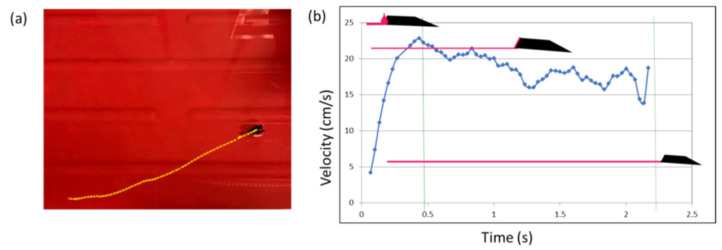
(**a**,**b**) When the boat was free to move over a bigger surface, we observed the propulsion from one side to the opposite.

Using the Tracker.jar software, we were able to elaborate the images and compute the boat position, determining a linear trajectory for the movement of the floating object and for the formation of the polymeric film. The results related to the motion tracking of Supplementary Video S6 are illustrated in Figure 8. The plot in Figure 8b displays the performance of the boat in terms of velocity. The boat moves along the horizontal plane *x* for 35 cm in a time of about 2 s. The velocity reached the maximum value of about 25 cm/s with a fast acceleration at the start (time 0) of about 100 cm^2^/s. The acceleration was maximum at the start of the motion because of its geometry; in fact, almost all the volume of the fuel drop came in contact with the water surface at the beginning. As a consequence, the boat moved, and then gradually, the residual volume of the polymeric solution glided on the water surface, retaining the locomotion with a mean velocity of 18 cm/s and a mean acceleration of 20 cm^2^/s. Once the residual volume ended, the mini-boat stopped.

### 3.3. Case of Use: Propulsion of Multiple Objects and Moving away Floating Particles

**Figure 9:**
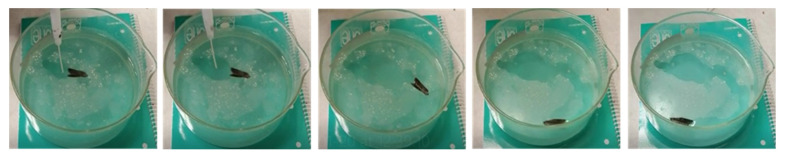
The locomotion and formation of the polymeric layer work even in the case of a uniform monolayer film created using commercial talc powder and move the powder away, cleaning the corresponding area.

The remover property related to on-the-fly formation of a polymeric film ejected from a floating boat is here proved in the case of a uniform and monolayer film created using commercial talc powder. In the frames shown in Figure 9, it is possible to observe the locomotion and film formation activated by drop dispensing. The boat moved the particles away during its locomotion, giving space to the formation of the polymeric layer. The activation of the polymer-spreading process led to the formation of a thin and transparent film that, until it was removed, moved the talc film away, cleaning the water surface and making it transparent. Once the film was recovered, the talc powder was free to cover the entire water surface. The presence of the talc powder could make the visualization of the thin film formed by the boat easier.

**Figure 10:**
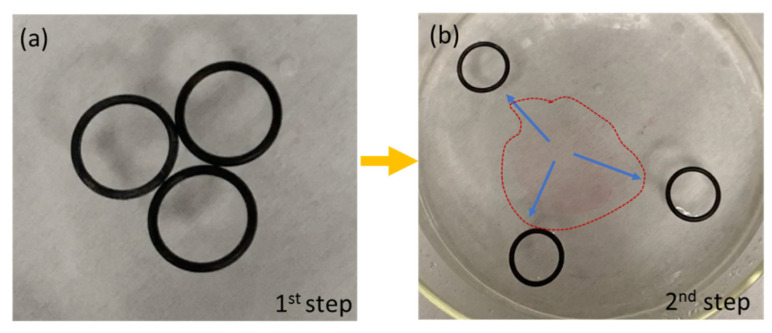
(**a**) Polymer droplets are dispensed in the central area, delimited by the points of contact of the three black o-rings; (**b**) the instantaneous formation of the film moves the floating objects away, inducing propulsion of multiple objects at the same time. On-the-fly formed polymer film is indicated by the dotted line.

In addition, the formation of the polymeric film could be used for the removal and passive locomotion of objects floating on the water surface without contact. In the case of Figure 10, three rubber o-rings were floating over the water surface and in contact with each other in a triangular configuration. The polymeric solution was dispensed on the central area, delimited by the points of contact of the three o-rings. When the polymer drop came into contact with the water, the instantaneous formation of the film took place, while the three o-rings were pushed away along three different directions. An interesting aspect is that the floating objects remained interconnected by the polymer layer, i.e., the on-the-fly membrane was able to capture floating objects, thus suggesting an interesting strategy for collecting and facilitating the water’s surface cleaning from floating dispersed plastics. In the case of locomotion combined with on-the-fly film formation, the cleaning procedure could cover different areas of the water surface reached by the object used as a locomotor. The propulsion of multiple objects during the formation of the polymeric thin film is visibly shown in Supplementary Video S7. In a case of interest, the polymer film could be collected from the water as a free-standing layer and transferred directly to the device of interest, as shown and characterized in the static configuration [32].

## 4. Discussion

In summary, we have developed an environmentally friendly, cost-effective water-based bottom-up approach in which floating objects driven by the Marangoni effect eject films on the fly while moving on the water surface. We have proved the self-rotation, free and/or along a pivot, of common objects without further effort in their design and functionalization. By controlling the area available for the spreading of the polymer droplets, we can shift from rotation to propulsion of the floating objects along a linear or circular path. We provided evidence for the first time of the propulsion and running formation of thin polymeric films by the activation of the Marangoni effect due to the diffusion of the solvent in water. It would be possible to tune the rotation and/or the propulsion, controlling the total amount of droplets dispensed (volume) to measure the spreading, the kinetics, and the viscoelastic properties of interfacial layers, and investigating different kinds of solvents and multicomponent droplets, but this characterization is beyond the focus of the present work, which provides a proof of concept of the principle for the target purpose. In fact, here, we want to furnish evidence of a new approach, where, by dispensing polymeric solution droplets, it is possible to propel, control the motion, and create on-the-fly polymeric layers for any class of floating object made of different shapes and materials, such as metal and plastics of different dimensions. All the experiments were conducted using tap water in a basic configuration. We also reported the propulsion characterization, various strategies for its implementation, and some cases of use for the as-formed thin films, such as the propulsion of multiple objects and moving away or capturing particles. The cleaning property could, in the future, be adopted for recovering dispersed micro-plastics, or in case of surface modification, the as-formed film could even chemically bind residual elements in water. In fact, it would be very interesting to design a guided locomotion that could be guided towards polluted zones and activate chemical reactions to bind and recover pollutants. Based on the principle of operation described here, we foresee the application of the new method proposed for developing new robotic and intelligent systems working on water at different scales, where the friction of the water surface could be tuned by adding commercial surfactants for further exploitation. This work is still a foundational study, which integrated a microfluidic device into a liquid interface-traveling system, but the formation of polymer thin films on the water surface can find a variety of useful applications in many fields, such as biochemistry, biotechnologies, environmental purposes, or packaging of food and beverages. Future investigation is required for its potential applications, such as remote sensing systems for aqueous environments, environmental remediation of a liquid interface, or on-site chemical reactions.

## 5. Conclusions

In conclusion, we have proposed a new approach where a single polymer drop can induce the Marangoni-driven self-propulsion of common macro-objects floating on water. At the same time, we demonstrated that it can drive the formation “on the fly” of a thin polymer film at the water surface. The same solvent used for the preparation of the polymeric solution and the film formation is used at the same time for the creation of a surface gradient able to induce the self-propelling of the object. Controlling the position where the droplet is delivered and the total amount of the dispensed volume, it is possible to tune the size, shape, and extension of the formed film. In fact, we demonstrated that by using polymer droplets with an appropriate concentration of solvent and delivering such drops at specific locations onto freely floating objects, it is possible to form “on-the-fly” thin polymer films at the free water surface. The self-rotation, free and/or along a pivot, of common objects without further effort in their design and functionalization was demonstrated, as was a shift from rotation to propulsion of the floating objects along a linear or circular path by controlling the area available for the spreading of the polymer droplets. In addition, the formation of the polymeric thin film for the removal and passive locomotion of objects floating on the water surface without contact was presented as well. By mixing self-propulsion and film formation, the achieved polymer films can cover quite extensive areas with different shapes depending on the motion of the floating object. This study suggests that characteristic features of Marangoni self-propelled motion could find applications ranging from the biological to technological worlds.

## Data Availability

Data is contained within the article or supplementary material.

## Supplementary Materials

The following supporting information can be downloaded at: https://www.mdpi.com/article/10.3390/polym14153228/s1, Video S1: On-the-fly thin film formation over water surface. Video S2: Rotation of Al square grid around a pivot. Video S3: Thin film formed on the fly by a floating plastic tube. Video S4: Propulsion and film formation by a floating boat. Video S5: Propulsion and film formation along a linear path. Video S6: Propulsion and film formation analyzed by Tracker.jar software. Video S7: Propulsion of multiple objects.
